# Age-Related Eye Diseases in Individuals With Mild Cognitive Impairment and Alzheimer's Disease

**DOI:** 10.3389/fnagi.2022.933853

**Published:** 2022-07-14

**Authors:** Jacqueline Chua, Zheting Zhang, Damon Wong, Bingyao Tan, Bhavani Kulantayan, Chelvin C. A. Sng, Saima Hilal, Narayanaswamy Venketasubramanian, Boon Yeow Tan, Carol Y. Cheung, Gerhard Garhöfer, Alina Popa-Cherecheanu, Tien Yin Wong, Christopher Li-Hsian Chen, Leopold Schmetterer

**Affiliations:** ^1^Singapore National Eye Centre, Singapore Eye Research Institute, Singapore, Singapore; ^2^Ophthalmology and Visual Sciences Academic Clinical Program, Duke-NUS Medical School, National University of Singapore, Singapore, Singapore; ^3^SERI-NTU Advanced Ocular Engineering (STANCE), Singapore, Singapore; ^4^Lee Kong Chian School of Medicine, Nanyang Technological University, Singapore, Singapore; ^5^School of Chemical and Biomedical Engineering, Nanyang Technological University, Singapore, Singapore; ^6^Department of Ophthalmology, National University of Singapore, Singapore, Singapore; ^7^Departments of Pharmacology and Psychological Medicine, Memory Aging and Cognition Centre, Yong Loo Lin School of Medicine, National University of Singapore, Singapore, Singapore; ^8^Saw Swee Hock School of Public Health, National University of Singapore and National University Health System, Singapore, Singapore; ^9^Raffles Neuroscience Centre, Raffles Hospital, Singapore, Singapore; ^10^St. Luke's Hospital, Singapore, Singapore; ^11^Department of Ophthalmology and Visual Sciences, The Chinese University of Hong Kong, Hong Kong, China; ^12^Department of Clinical Pharmacology, Medical University Vienna, Vienna, Austria; ^13^Carol Davila University of Medicine and Pharmacy, Bucharest, Romania; ^14^Department of Ophthalmology, Emergency University Hospital, Bucharest, Romania; ^15^Center for Medical Physics and Biomedical Engineering, Medical University Vienna, Vienna, Austria; ^16^Institute of Molecular and Clinical Ophthalmology, Basel, Switzerland

**Keywords:** age-related macular degeneration, diabetic retinopathy, cognitive impairment no dementia, Alzheimer's disease, dementia

## Abstract

**Introduction:**

Alzheimer's disease (AD) and age-related eye diseases pose an increasing burden as the world's population ages. However, there is limited understanding on the association of AD/cognitive impairment, no dementia (CIND) with age-related eye diseases.

**Methods:**

In this cross-sectional, memory clinic-based study of multiethnic Asians aged 50 and above, participants were diagnosed as AD (*n* = 216), cognitive impairment, no dementia (CIND) (*n* = 252), and no cognitive impairment (NCI) (*n* = 124) according to internationally accepted criteria. Retinal photographs were graded for the presence of age-related macular degeneration (AMD) and diabetic retinopathy (DR) using standard grading systems. Multivariable-adjusted logistic regression models were used to determine the associations between neurological diagnosis and odds of having eye diseases.

**Results:**

Over half of the adults had at least one eye disease, with AMD being the most common (60.1%; *n* = 356), followed by DR (8.4%; *n* = 50). After controlling for age, sex, race, educational level, and marital status, persons with AD were more likely to have moderate DR or worse (OR = 2.95, 95% CI = 1.15–7.60) compared with NCI. In the fully adjusted model, the neurological diagnosis was not associated with AMD (OR = 0.75, 95% CI = 0.45–1.24).

**Conclusion:**

Patients with AD have an increased odds of having moderate DR or worse, which suggests that these vulnerable individuals may benefit from specific social support and screening for eye diseases.

## Introduction

The proportion of older individuals is rising rapidly, particularly in developed countries. Globally, ~2.1 billion people are estimated to be aged 60 and older in 2050 (United Nations Department of Economic Social Affairs Population Division, 2017). Aging causes a variety of changes to the brain and eye, making an older person more prone to Alzheimer's disease (AD) and visual impairment. Both AD (Wood et al., 2016) and eye diseases (Knudtson et al., 2005) decrease quality of life and worsen active aging. The major age-related eye diseases, including age-related macular degeneration (AMD) and diabetic retinopathy (DR), are the leading causes of visual impairment and blindness worldwide (Flaxman et al., 2017). In the light of the rising incidence of both AD (GBD 2016 Dementia Collaborators, 2019, 2021) and age-related eye diseases (Tham et al., 2014; Wong et al., 2014; Teo et al., 2021), knowledge on the pattern of age-related eye diseases in individuals with AD and cognitive impairment, no dementia (CIND) (Jacova et al., 2008) is critical and useful in the context of screening, early detection, and management of such ophthalmic conditions in cognitively impaired individuals.

In recent years, several studies have investigated the influence of neurological diagnosis on AMD but with inconclusive results (Baker et al., 2009; Keenan et al., 2014; Nolan et al., 2014; Williams et al., 2014; Frost et al., 2016). While Frost et al. found an increased prevalence of early AMD in patients with AD compared to cognitively normal controls (Frost et al., 2016), other researchers reported no association between AD and AMD after adjusting for potential confounders (Baker et al., 2009; Nolan et al., 2014; Williams et al., 2014). Contrary to previous studies which were cross-sectional in study design, a longitudinal study by Keenan et al. (2014) found that dementia and AD were associated with *decreased* incidence of AMD. However, their study was derived from patients seeking tertiary eye care services, which may represent the more severe spectrum of the disease, admission to hospital for AMD treatments, i.e., intravitreal antivascular endothelial growth factor (VEGF) therapy. Overall, past studies focused overwhelmingly on western populations, reflecting a paucity of data on other races/ethnicities. While several studies have examined the impact of having DR on cognitive impairment (Cheng et al., 2021), no studies have examined the impact of having cognitive impairment on DR. Finally, very limited data are available on the impact of CIND on eye diseases, a condition with established risk of progression to AD and other forms of dementia (Petersen, 2016).

In this study, we examined the associations of neurological diagnosis with age-related eye diseases, namely, AMD and DR, in an Asian setting. We hypothesized that prevalence of eye diseases will be higher in persons with cognitive impairment than NCI.

## Materials and Methods

### Study Participants

We conducted a memory-clinic-based cross-sectional study, approved by the National Healthcare Group Domain-Specific Review Board (protocol number R1500/83/2017), and conducted in accordance with the Declaration of Helsinki. Written informed consent was obtained from all the participants or their caregivers prior to the recruitment for this study. Individuals aged 50 and older were recruited from the National University Hospital of Singapore and St Luke's Hospital from September 2009 to September 2020, as described previously (Chua et al., 2020). Subjects with no cognitive impairment (NCI) were recruited from both memory clinics and the community. The etiological diagnoses of dementia were based on internationally accepted criteria: Alzheimer's disease (AD) was diagnosed using the National Institute of Neurological and Communicative Disorders and Stroke and the Alzheimer's Disease and Related Disorders Association (NINCDS-ADRDA) (McKhann et al., 2011); vascular dementia (VaD) was defined using the National Institute of Neurological Disorders and Stroke and Association Internationale pour la Recherché et l'Enseignement en Neurosciences (NINDS-AIREN) criteria (Román et al., 1993). Cognitive impairment with no dementia (CIND) was determined based on the objective impairment in at least one domain of the neuropsychological assessment but did not meet the DSM-IV criteria for dementia. Mild cognitive impairment (MCI) was defined using the clinical dementia rating (CDR) scale, where scoring 0.5 on the CDR among participants with CIND will fulfill the criteria of subjective cognitive complaints (Morris, 2012). Specifically, to score 0.5 on the CDR, the participant has responded “yes” to the question “Have you had any problem with your thinking or memory?” or a caregiver has responded “yes” to the question “Does he/she have a problem with his/her memory or thinking?” Thinking problems included complaints related to executive dysfunction (e.g., poor organization, distractible, and difficulty with problem-solving) and visuospatial impairment (e.g., does not recognize familiar landmarks). Participants were classified as NCI if they had no objective impairment in any of the seven domains of the neuropsychological assessment.

Participants were excluded from this study if they had clinically relevant conditions that would significantly impede cognitive assessment and self-reported diagnosis of glaucoma due to the impending risks of acute angle-closure glaucoma with mydriatic eyedrops. All participants underwent detailed clinical and neuropsychological assessments (Gyanwali et al., 2019). Trained research psychologists administered brief cognitive tests: the Mini-Mental State Examination (MMSE) and the Montreal Cognitive Assessment (MoCA) and a formal detailed neuropsychological test battery that has been locally validated in Singapore (Hilal et al., 2021).

### Demographics and Vascular Risk Factors

Information on participants' demographic, socioeconomic characteristics (e.g., age, sex, education, marital status [married, single, divorced, and widowed] (Zheng et al., 2014), and living situation [living with partner/spouse, living with children/relative/friend, living alone and other forms of arrangement]), smoking, and medical history (e.g., hyperlipidemia, hypertension, and diabetes) was collected using a standardized questionnaire and subsequently verified by medical records. Education was recorded as the highest number of years of schooling completed and was categorized into 2 groups: (1) Primary or lower (≤ 6 years, equivalent to elementary education) and (2) secondary or higher (≥7 years, equivalent to high school or college, including university education). Height was measured in centimeters using a wall-mounted measuring tape and weight in kilograms using a digital scale. Body mass index (BMI), is calculated as body weight (in kilograms) divided by body height (in meters) squared. Seated blood pressure measurements were taken using an automated oscillometric device during their clinical visits.

### Definition of Eye Diseases

**Figure 1:**
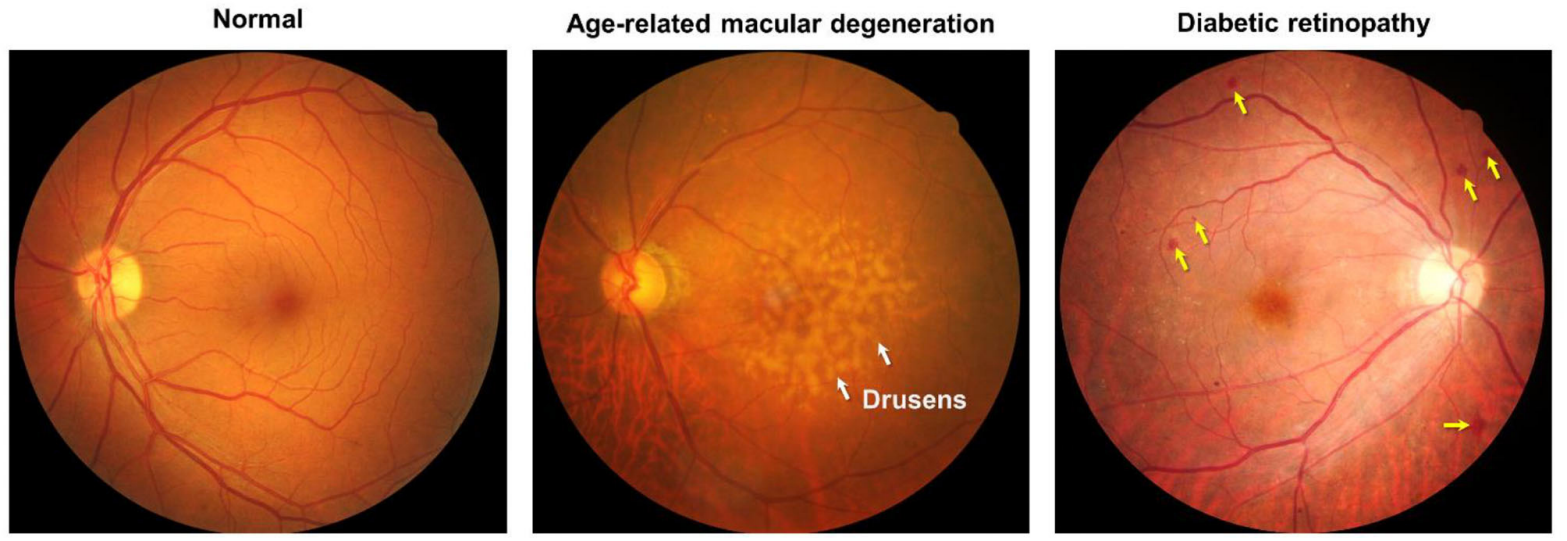
(Left panel) Color fundus photograph of the left eye without any eye diseases. (Middle panel) Color fundus photograph of the left eye with signs of intermediate age-related macular degeneration (AMD). Notably, features include large soft drusen at the macular region (white arrows), (Right panel) Color fundus photograph of the right eye with signs of severe non-proliferative diabetic retinopathy (DR). Notably, features include microaneurysms and hemorrhages (yellow arrows).

Ocular assessments were performed within 1 month of the cognitive assessments. Digital retinal photographs were taken using a nonmydriatic camera (Canon CR-1 Mark II, Canon, Ota, Tokyo, Japan), after pupil dilation using tropicamide 1%. For each patient, two retinal photographs (optic disc and fovea) were taken of each eye. One trained grader masked to the participant's characteristics assessed the fundus photographs for the presence of age-related macular degeneration (AMD) and diabetic retinopathy (DR) (Figure 1) (Wilkinson et al., 2003; Davis et al., 2005). If more than one-quarter of the photograph was obscured, it was considered ungradable. AMD was defined according to the Age-Related Eye Disease Study grading system, where early AMD was defined as drusen outside 2-disc diameters (2DD) of the macula center or drusen within 2DD but ≤125 μm in greatest linear diameter, intermediate AMD as numerous medium-sized drusen, 1 large drusen >125 μm in greatest linear diameter, non-central geographical atrophy, and advanced AMD as central geographical atrophy or neovascular AMD (Davis et al., 2005). Diabetic retinopathy was defined as a severity level of moderate non-proliferative diabetic retinopathy (NPDR) or worse, and/or diabetic macular edema (defined as the presence of hard exudates, microaneurysms, and hemorrhages at the posterior pole of the retinal images) using the International Classification Diabetic Retinopathy Scale (Wilkinson et al., 2003). We further categorized other types of retinal pathologies, i.e., macular hole, retinal vascular occlusives, etc.

### Statistical Analyses

The primary outcome of the study was eye disease. The Shapiro–Wilk test was used to assess the normality of the distribution of the continuous variables. To compare the characteristics of participants among groups, independent *t*-test or one-way analysis of variance (ANOVA) was performed for continuous variables, and chi-square or Fisher's exact tests were performed for categorical variables. Age-, sex-, and ethnicity-adjusted and multivariable-adjusted logistic regression models were used to determine the odds ratios (ORs) and 95% confidence intervals (CIs) for associations between neurological diagnosis (exposure) and age-related eye diseases (outcome). The potential confounders we considered were age, sex, ethnicity, education attainment, marital status, living situation, hypertension, and systolic blood pressure. Statistically significant confounders were determined using manual backward elimination procedures with a criterion of *p* < 0.10 for elimination. A *p* < 0.05 was considered statistically significant. Statistical analyses were performed using SPSS Statistics version 26 (IBM) and STATA, version 16; StataCorp LP.

## Results

A total of 656 participants were recruited between September 2009 to September 2020, 592 (90.2%) of whom had gradable fundus photographs. A comparison of characteristics between persons with and without gradable fundus photographs is shown in Supplementary Table 1. Persons who had ungradable photographs were more likely to be older and have AD and diabetes (*p* ≤ 0.034). A total of 124 NCI participants, 252 CIND participants, and 216 AD participants with gradable fundus images were available for analysis.

Table 1 presents the characteristics of the participants with gradable fundus photographs by their neurological diagnosis. The mean ± standard deviation (SD) of age was 68.3 ± 7.7 years for NCI, 72.8 ± 7.7 years for CIND, and 74.7 ± 7.4 years for AD. AD participants were likely to be older and of Malay ethnicity, had lower educational level, more likely to be divorced and widowed, more likely to be living with children, relatives, or friends, higher prevalence of hypertension and diabetes, and higher systolic blood pressure (*p* ≤ 0.007). There was no significant difference in sex, smoking status, hyperlipidemia, BMI, and diastolic blood pressure among the groups.

**Table 1:** Characteristics of participants stratified by neurological diagnosis.

**Characteristics**	**NCI (*n* = 124)**	**CIND (*n* = 252)**	**AD (*n* = 216)**	***P*** -value^*^
Age, years	68.3 (7.7)	72.8 (7.7)	74.7 (7.4)	**<0.001**
**Sex**				
Male	56 (45.2)	118 (46.8)	86 (39.8)	0.298
Female	68 (54.8)	134 (53.2)	130 (60.2)	
**Ethnicity**				
Chinese	115 (92.7)	209 (82.9)	170 (78.7)	**0.007**
Malay	3 (2.4)	20 (7.9)	31 (14.4)	
Indian	5 (4.0)	19 (7.5)	11 (5.1)	
Others	1 (0.8)	4 (1.6)	4 (1.9)	
**Educational level**				
Primary or lower	39 (31.5)	121 (48.0)	151 (69.9)	**<0.001**
Secondary or higher	85 (68.5)	131 (52.0)	65 (30.1)	
**Marital status**				
Married	101 (81.5)	176 (69.8)	136 (63.0)	**<0.001**
Single	10 (8.1)	15 (6.0)	6 (2.8)	
Divorced	3 (2.4)	7 (2.8)	10 (4.6)	
Widowed	10 (8.1)	54 (21.4)	64 (29.6)	
**Living situation**				
Lives with partner/spouse	100 (80.6)	169 (67.1)	114 (52.8)	**<0.001**
Lives with children/relative/friend	9 (7.3)	60 (23.8)	85 (39.4)	
Lives alone	12 (9.7)	11 (4.4)	8 (3.7)	
Others	3 (2.4)	12 (4.8)	9 (4.2)	
**Smoking^†^**				
Current smoker	11 (8.9)	17 (6.7)	14 (6.5)	0.448
Ex-smoker	17 (13.7)	50 (19.8)	47 (21.8)	
Non-smoker	95 (76.6)	185 (73.4)	155 (71.8)	
**Hyperlipidemia^‡^**				
Yes	82 (66.1)	184 (73.0)	154 (71.3)	0.323
No	42 (33.9)	66 (26.2)	62 (28.7)	
**Hypertension^§^**				
Yes	72 (58.1)	164 (65.1)	171 (79.2)	**<0.001**
No	52 (41.9)	86 (34.1)	43 (19.9)	
**Diabetes**				
Yes	19 (15.3)	84 (33.3)	91 (42.1)	**<0.001**
No	105 (84.7)	168 (66.7)	125 (57.9)	
Body mass index^‖^, kg/m^2^	24.2 (3.8)	24.0 (3.9)	23.7 (4.3)	0.423
Systolic blood pressure^¶^, mmHg	138.0 (17.5)	144.1 (17.6)	144.9 (21.5)	**0.004**
Diastolic blood pressure^#^, mmHg	73.0 (10.5)	74.9 (10.3)	72.9 (10.8)	0.075

*Data presented are mean (SD) or number (%), as appropriate*.

*NCI, no cognitive impairment; CIND, cognitive impairment, no dementia; AD, Alzheimer's disease*.

* *p-Value was obtained with one-way analysis of variance (ANOVA) for the continuous variables and with the chi-square test or Fisher's exact test for the categorical variables*.

*Bold values denote statistical significance at the p < 0.05 level*.

† *Data available for 123 NCI, 252 CIND, and 216 patients with AD*.

‡ *Data available for 124 NCI, 250 CIND, and 216 patients with AD*.

§ *Data available for 124 NCI, 250 CIND, and 214 patients with AD*.

‖ *Data available for 124 NCI, 250 CIND, and 214 patients with AD*.

¶ *Data available for 124 NCI, 250 CIND, and 216 patients with AD*.

# *Data available for 124 NCI, 249 CIND, and 216 patients with AD*.

**Figure 2:**
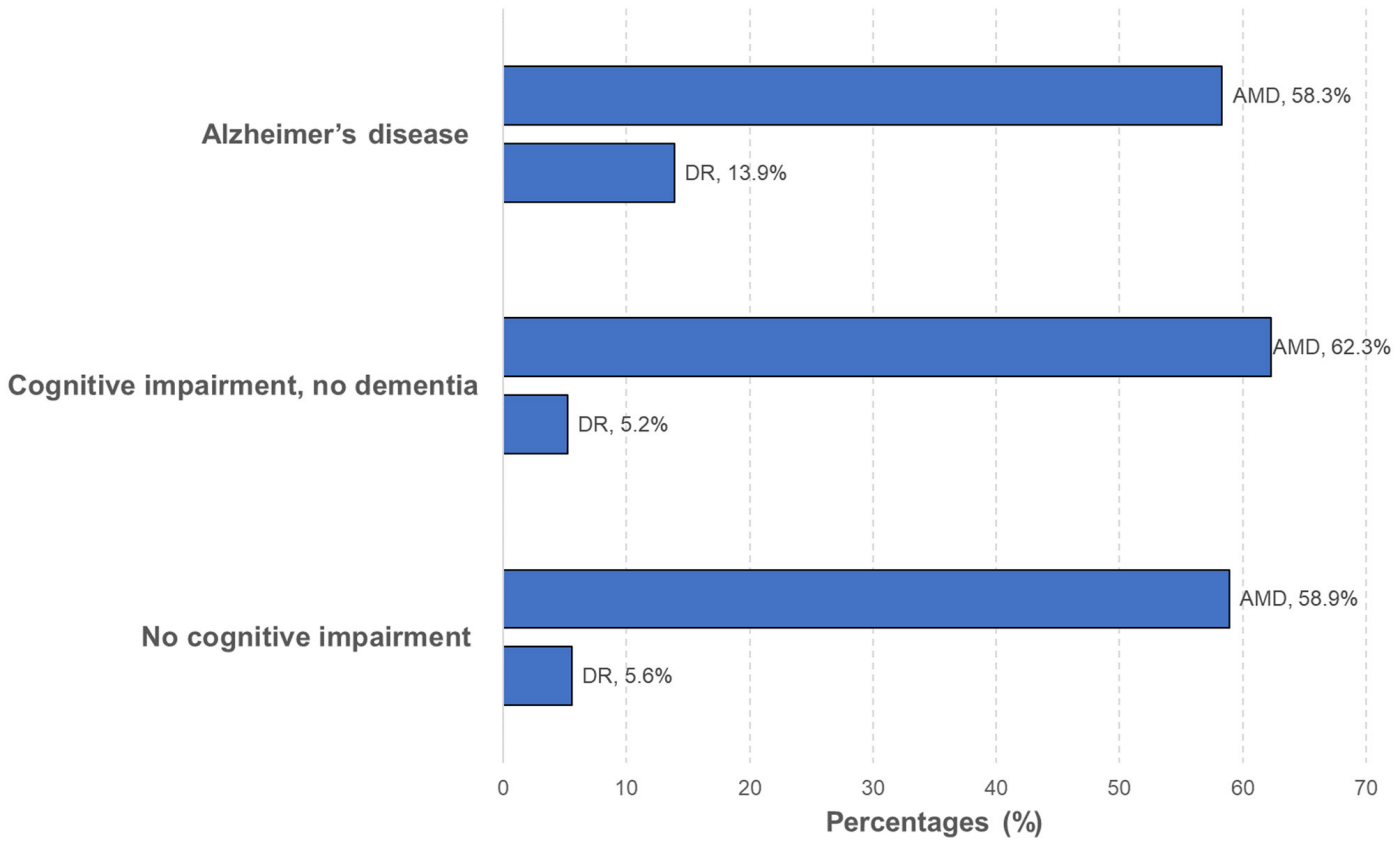
Types of eye diseases by neurological diagnosis. Diabetic retinopathy (DR) status was significantly higher in patients with Alzheimer's disease whereas the presence of age-related macular degeneration (AMD) was similar across the neurological groups.

Of the 592 participants with gradable photographs, 396 (66.9%) had at least one eye disease, with AMD being the most common (60.1%; *n* = 356), followed by DR (8.4%; *n* = 50). Table 2 shows the associations of neurological diagnosis with any eye diseases, AMD, and DR. Only AD participants were more likely to have higher odds of having DR (OR = 2.95, 95% CI = 1.15–7.60) in the multivariable model. However, this association was no longer significant after further adjusting for the presence of diabetes (OR = 1.83, 95% CI = 0.66–5.04). We next analyzed the association of DR in AD in persons with vascular dementia and report a stronger association between DR and AD (OR = 5.22, 95%CI = 1.74–15.67; Table 2). Neurological diagnosis was not significantly associated with an increased odds of having any AMD (OR = 0.75, 95%CI = 0.45–1.24; Table 2). Figure 2 further demonstrates that the presence of AMD was similar across the neurological groups whereas DR status was significantly higher in AD participants. We performed a separate analysis and report that both CIND without MCI (OR = 1.07, 95% CI = 0.29–3.97) and CIND with MCI (OR = 0.99, 95% CI = 0.35–2.82) were not independently associated with having DR (Supplementary Table 2). We also performed a subgroup analysis and report that both CIND and AD were not independently associated with either early AMD (OR = 0.83, 95% CI = 0.44–1.58) or intermediate AMD or worse (OR = 0.68, 95% CI = 0.38–1.22; Supplementary Table 3).

**Table 2:** Association between neurological diagnosis and eye diseases.

	**Eye diseases**			**AMD**			**Diabetic retinopathy**			
**Neurological diagnosis**	**No. (%)**	**Model 1**	**Model 2**	**No. (%)**	**Model 1**	**Model 2**	**No. (%)**	**Model 1**	**Model 2**	**Model 3**
NCI	80 (64.5)	Reference	Reference	73 (58.9)	Reference	Reference	7 (5.6)	Reference	Reference	Reference
CIND	170 (67.5)	0.98 (0.62–1.58)	0.94 (0.58–1.51)	157 (62.3)	0.96 (0.61–1.52)	0.95 (0.60–1.51)	13 (5.2)	1.09 (0.42–2.88)	1.01 (0.38–2.70)	0.66 (0.23–1.86)
AD	146 (67.6)	0.94 (0.57–1.56)	0.86 (0.51–1.44)	126 (58.3)	0.77 (0.47–1.25)	0.75 (0.45–1.24)	30 (13.9)	**3.60 (1.45–8.97)**	**2.95 (1.15–7.60)**	1.83 (0.66–5.04)
NCI	80 (64.5)	Reference	Reference	73 (58.9)	Reference	Reference	7 (5.6)	Reference	Reference	Reference
AD	115 (67.6)	0.94 (0.55–1.59)	0.86 (0.50–1.48)	103 (60.6)	0.84 (0.50–1.41)	0.83 (0.49–1.40)	19 (11.2)	**2.80 (1.07–7.34)**	2.27 (0.84–6.15)	1.43 (0.49–4.18)
Vascular dementia	31 (67.4)	0.96 (0.46–2.00)	0.85 (0.40–1.82)	23 (50.0)	0.57 (0.28–1.15)	0.55 (0.27–1.13)	11 (23.9)	**6.20 (2.13–18.01)**	**5.22 (1.74–15.67)**	2.90 (0.90–9.31)

*AMD, age-related macular degeneration; NCI, no cognitive impairment; CIND, cognitive impairment, no dementia; AD, Alzheimer's disease*.

*Model 1: Adjusted for age, sex, and ethnicity*.

*Model 2: Adjusted for age, sex, ethnicity, educational level, and marital status*.

*Model 3: Adjusted for age, sex, ethnicity, educational level, marital status, and diabetes*.

*Bold values denote statistical significance at the p < 0.05 level*.

## Discussion

In a multiethnic Asian population, our data showed that the odds are three times higher that an patient with AD will get moderate DR or worse compared to a participant with NCI whereas neurological diagnosis was not associated with AMD, after controlling for age, sex, race, educational level, and marital status. There is less available data comparing the odds of DR between cognitive groups. Further statistical adjustment for the presence of diabetes demonstrates that the association between DR and AD is due to higher numbers of diabetic individuals in the AD cohort compared with the CIND and NCI cohorts. A meta-analysis pooled a total of 17 studies involving 1,746,777 individuals reported that participants with diabetes had significant higher incidence of AD than those without diabetes (Zhang et al., 2017).

To our knowledge, this is the first study investigating the risk of DR in subjects with clinically diagnosed CIND or AD (Table 3). Previous studies investigating the association between DR and cognitive function all focused on DR as the independent factor, i.e., as a potential marker for present or incident CIND/AD. To this end, they found that patients with DR had increased prevalence (Ong et al., 2012; Liao et al., 2017) and incidence (Gupta et al., 2019) of CIND, as well as increased incidence of AD (Chen et al., 2016; Lee et al., 2019; Pedersen et al., 2022). A recent systematic review and meta-analysis support these results by concluding that having moderate DR or worse was strongly associated with cognitive impairment (Cheng et al., 2021). Considered in tandem with the findings of our study, this suggests that the association between DR and cognitive impairment may be bidirectional. Although there is consistent evidence of shared risk factors between retinal and cerebral neurodegeneration (Jindal, 2015; Ong et al., 2015), including microvasculature-specific associations (Chua et al., 2020), the contribution of AD in the pathogenesis of DR has not been elucidated. In particular, it has been suggested that vascular lesions in eyes with DR may mirror similar pathological processes in the cerebral microcirculation (Cheung and Wong, 2008). Our finding of increased odds of DR in vascular dementia supports this proposition. Future longitudinal studies will need to be performed to determine the risk of DR in pre-diabetic AD and CIND cases.

**Table 3:** Summary of studies investigating the association between Alzheimer's disease (AD) and eye diseases.

**First author, year**	**Country**	**Study design**	**Study population**	**Sample size**	**Diagnosis of AD**	**Outcome measure**	**Definition of AMD/DR**	**Confounders addressed through design or analysis**	**Association of AD with AMD/DR**
**Age-related macular degeneration**									
Frost et al. (2016)	Australia	Cross-sectional	AD: Participants with AD (all White Caucasians, ≥55 years old) from the Australian Imaging, Biomarkers and Lifestyle (AIBL) study of aging Control: Participants without AD from the same study	AD: 22 Control: 101	AD: Clinical diagnosis (NINCDS-ADRDA)	Prevalence of AMD: Retinal photography; Clinical diagnosis (Beckman classification)	No clinically relevant AMD: small drusen or no drusen, no pigmentary abnormalities Early AMD: medium drusen without pigmentary abnormalities Intermediate AMD: large drusen, or medium drusen with pigmentary abnormalities Late AMD: lesions associated with neovascular AMD or geographic atrophy	Adjusted: age, smoking, hypertension, high- and low-density lipoproteins, cataract surgery, APOE ε4 carrier status	AD was associated with increased prevalence of early AMD (OR 18.67, 95%CI 4.42–78.80, *P* < 0.0001) when adjusted for confounders.
Nolan et al. (2014)	Ireland	Cross-sectional	AD: Patients with mild to moderate AD (predominantly moderate) attending the Age-Related Care Unit at Waterford Regional Hospital, Waterford, Ireland Control: Subjects recruited *via* newspaper and radio advertisements and word of mouth, spouses of subjects with AD	AD: 36 Control: 33	Mild to moderate AD: Clinical diagnosis (MMSE score 14–24 with documented difficulties carrying out everyday tasks, and some alteration in behavior)	Macular pigment (MP): Central MP at 0.23° eccentricity, MP volume (Heidelberg Spectralis® HRA+OCT Multicolor) Visual function: Best corrected visual acuity (BCVA), contrast sensitivity (CS) (computerized LogMAR ETDRS test chart) Prevalence of early AMD: Retinal photography; Clinical diagnosis (International Classification and Grading System for AMD)	Early AMD: presence of soft drusen and/or hypo-/hyper-pigmentary changes at the macula	Adjusted: age, diet, education, macular pigment	AD was not associated with increased prevalence of AMD when adjusted for confounders.
Williams et al. (2014)	United Kingdom	Cross-sectional	AD: Patients ≥65 years old opportunistically recruited in clinic Control: Respondents to press release, friends of controls, carers of patients, patient support groups	AD: 258 Control: 322	AD: Clinical diagnosis (NINCDS-ADRDA)	Prevalence of AMD: Retinal photography; Clinical diagnosis (“Whitla grades”)	Grade 0: no signs of age-related maculopathy; or hard drusen (<63 μm diameter) only Grade 1: soft distinct drusen (>63 μm) only; or pigment abnormalities only Grade 2: soft indistinct drusen (>125 μm) or reticular drusen only; soft distinct drusen (>63 μm) with pigment abnormalities; or soft indistinct drusen (>125 μm) or reticular drusen with pigment abnormalities Grade 3: geographic atrophy; or neovascular AMD	Adjusted: age, smoking, ‘generally unwell recently’, APOE ε3ε4, APOE ε4ε4	AD was not associated with increased prevalence of AMD when adjusted for confounders.
Baker et al. (2009)	United States	Cross-sectional	Low DSST/Low 3MSE/Dementia/AD: Participants with low DSST/low 3MSE/dementia/AD from the Cardiovascular Health Study, recruited from a random sample of Medicare eligibility lists from 4 US counties Reference: Participants without low DSST/low 3MSE/dementia/AD from the same study	Low DSST: 443 Reference: 1472 Low 3MSE: 481 Reference: 1487 Dementia: 135 Reference: 1278 AD: 86 Reference: 1278	Low DSST: Lowest quartile of DSST scores (≤ 30) Low 3MSE: Lowest quartile of 3MSE scores (≤ 89) Dementia: Clinical diagnosis (DSM-IV) AD: Clinical diagnosis (DSM-IV)	Prevalence of early AMD: Retinal photography; Clinical diagnosis (modification of the Wisconsin AMD grading system)	Early AMD: presence of soft drusen alone, retinal pigment epithelial depigmentation alone, or a combination of soft drusen with increased retinal pigment or depigmentation in the absence of late AMD Late AMD: presence of exudative AMD (subretinal hemorrhage, subretinal fibrous scar, retinal pigment epithelial detachment, or serous detachment of the sensory retina) or pure geographic atrophy	Adjusted: age, sex, ethnicity, study center, education (completed high school), systolic blood pressure, total cholesterol level, diabetes, smoking status, apolipoprotein E (6 genotypes)	Low DSST score (≤ 30) was associated with increased prevalence of early AMD (OR 1.38, 95% CI 1.03–1.85) when adjusted for confounders. 3MSE score, dementia, or AD were not associated with increased prevalence of early AMD.
Keenan et al. (2014)	United Kingdom	Cohort	Dementia: Patients ≥50 years old with an admission or day case care for dementia, constructed from the English National Health Service from January 1, 1999 to February 28, 2011 Reference: Individuals without dementia, constructed from the same source	Dementia: 168,092 Reference: >7.7 million	Dementia: Hospital computerized records (unspecified criteria)	Incidence of AMD: Hospital computerized records (unspecified criteria)	AMD: diagnosis recorded in computerized records (criteria not specified)	Adjusted: sex, age in 5-year bands, calendar year of admission, region of residence, Index of Multiple Deprivation score associated with patients' area of residence	Dementia (RR 0.07, 95% CI 0.04–0.11) and AD (RR 0.04, 95% CI 0.01–0.10) were associated with decreased incidence of admission for AMD when adjusted for confounders.
Current study	Singapore	Cross-sectional	AD: Patients ≥50 years old recruited from memory clinics at the National University Hospital and St Luke's Hospital, Singapore VaD: Patients from the same clinics CIND: Patients from the same clinics NCI: Individuals without cognitive impairment from the same clinics and the community	AD: 216 CIND: 252 NCI: 124	AD: Clinical diagnosis (DSM-IV and NINCDS-ADRDA criteria) VaD: Clinical diagnosis (NINDS-AIREN criteria) CIND: Clinical diagnosis (objective impairment in at least one domain of the neuropsychological assessment, but did not meet the DSM-IV criteria for dementia)	Prevalence of AMD: Retinal photography using the Age-Related Eye Disease Study grading system	Early AMD: drusen outside 2-disc diameters (2DD) of the macula center, or drusen within 2DD but ≤ 125 μm in greatest linear diameter Intermediate AMD: numerous medium-sized drusen, 1 large drusen >125 μm in greatest linear diameter, noncentral geographical atrophy Advanced AMD: central geographical atrophy or neovascular AMD	Adjusted: age, sex, race, educational level, marital status	Both AD and CIND were not associated with increased prevalence of AMD when adjusted for confounders.
**Diabetic retinopathy**									
Current study	Singapore	Cross-sectional	AD: Patients ≥50 years old recruited from memory clinics at the National University Hospital and St Luke's Hospital, Singapore VaD: Patients from the same clinics CIND: Patients from the same clinics NCI: Individuals without cognitive impairment from the same clinics and the community	AD: 216 CIND: 252 NCI: 124	AD: Clinical diagnosis (DSM-IV and NINCDS-ADRDA criteria) VaD: Clinical diagnosis (NINDS-AIREN criteria) CIND: Clinical diagnosis (objective impairment in at least one domain of the neuropsychological assessment, but did not meet the DSM-IV criteria for dementia)	Prevalence of DR: Retinal photography; Clinical diagnosis (International Classification of Diabetic Retinopathy)	DR: severity level of moderate non-proliferative diabetic retinopathy (NPDR) or worse, and/or diabetic macular edema (presence of hard exudates, microaneurysms and hemorrhages at the posterior pole)	Adjusted: age, sex, race, educational level, marital status	AD was associated with increased prevalence of DR (OR 2.95, 95% CI 1.15–7.60) when adjusted for confounders. CIND was not associated with increased prevalence of DR when adjusted for confounders.

*AMD, age-related macular degeneration; DR, diabetic retinopathy; AD, Alzheimer's disease; VaD; vascular dementia; CIND, cognitive impairment, no dementia; NCI, no cognitive impairment; NINCDS-ADRDA, National Institute of Neurological and Communicative Diseases and Stroke/Alzheimer's Disease and Related Disorders Association criteria for Alzheimer's disease; MMSE, Mini-Mental State Examination; 3MSE, Modified Mini-Mental State Examination; DSST, Digit Symbol Substitution Test; DSM-IV, Diagnostic and Statistical Manual of Mental Disorders, 4th Edition; DSM-IV-TR, Diagnostic and Statistical Manual of Mental Disorders, 4th Edition, Text Revision; NINDS-AIREN, National Institute of Neurological Disorders and Stroke-Association Internationale pour la Recherche et l'Enseignement en Neurosciences; ETDRS, Early Treatment Diabetic Retinopathy Study; OR, odds ratio; CI, confidence interval*.

There is currently no published guideline on the prevalence, management, and outcomes of DR in patients with AD, although there is a national diabetic retinal photograph screening for individuals with diabetes in Singapore (Goh et al., 2014). The reasons underpinning higher odds of DR in patients with AD are multifactorial. Apart from the higher prevalence of diabetes as pointed out earlier, individuals with AD may be less likely to attend annual DR screening program since they might be more reliant on caregivers to attend primary eyecare appointments and less likely to notice or report visual symptoms. Previous research reported that a high proportion of individuals with dementia are living with undiagnosed age-related eye diseases (Wong et al., 2015). A persistent lack of referral of moderate DR or worse can lead to worsening of retinopathy, visual impairment, or blindness, whereas timely delivery of sight-saving treatments can decrease the risk of blindness (Ting et al., 2017).

The finding that AD was not associated with AMD is in concordance with most studies on older White populations (Table 3) (Baker et al., 2009; Nolan et al., 2014; Williams et al., 2014). While an Australian study found increased prevalence of early AMD in subjects with AD (Frost et al., 2016), they did not find a significant association of AD diagnosis with intermediate and advanced AMD. It most likely indicates that AMD is associated with higher risks of cognitive dysfunction or AD, as consistently demonstrated in cross-sectional (Wong et al., 2002; Seden et al., 2015) and cohort (Klaver et al., 1999; Tsai et al., 2015; Chen et al., 2016; Lee et al., 2019; Choi et al., 2020) studies, but not *vice versa*. Another potential reason for the lack of association may have been consequent to the limited sample size and thus the lack of power in statistical analysis; to date, the odds of AMD in persons CIND or AD have not been investigated in a large-scale cohort study.

Notably, high prevalence of AMD (59.3%) was observed in adults aged 50 and older in this study. A previous population-based cross-sectional study of a multiethnic Asian cohort residing in Singapore found the prevalence of AMD (early and late AMD combined) in persons aged 80 and above to be 26.3% (Cheung et al., 2014). This difference may be accounted for partly by the different grading criteria used. The aforementioned population-based study used the modification Wisconsin Age-Related Maculopathy Grading System, which defines AMD as either soft indistinct or reticular drusen or both soft, distinct drusen plus retinal pigment epithelium abnormalities which is stricter than our study. Moreover, the AMD prevalence in our study of 59.3% is comparable to the 41.5% reported by Williams et al. (2014), similarly a clinic-based study in which disease prevalence would tend to be higher than population-based studies.

### Clinical Implications

Older people with AD are “vulnerable patients.” General physicians should highlight the importance of having regular diabetic eye screening to the patients and their caregivers. If this vulnerable group of people is not accessing screening appointments, this could lead to irreversible loss of vision (Cheung et al., 2010). Also, there is the question of what happens to patients with AD who have unsuccessful screening, either because of difficulties with retinal photography or because of a positive screen. In this study, we found that AD persons were more likely to have ungradable retinal photographs than their counterparts. Future studies need to evaluate the prevalence, management, and outcomes of DR screening services in AD individuals.

### Strengths and Limitations

Strengths of this study include the use of internationally recognized criteria for the clinical assessment of neurological status, objective grading of age-related eye diseases using fundus photographs, and the standardized assessment of other systemic and ocular factors. This study also has limitations. First, although the observed associations were statistically significant, the CIs were quite wide. Therefore, the clinical application of our findings needs to be further validated in future studies. Second, the cross-sectional nature of this study limits causal inferences. Further robust longitudinal studies are required to investigate this association. Third, although our study population comprises Asians, the cultural, health, and living conditions in Singapore are different from other Asian countries. Thus, our findings may not be entirely inferred to other Asian countries with the same ethnicity group. Moreover, as most study subjects were Chinese, we were not able to determine whether similar findings are seen in the Singaporean Malays or Indians. Fourth, we did not assess the association of neurological diagnosis on having glaucoma which is one of the common age-related eye diseases. Patients with glaucoma were excluded from the study as the use of pharmacological dilation drops may induce an episode of angle-closure glaucoma in susceptible elderly individuals. Furthermore, we recruited study participants from specialized memory disorder clinics and not from the population which may be subjected to bias and not as generalizable. Finally, in our study, AD individuals tended to have ungradable photographs as compared to CIND or NCI individuals, and this might have resulted in potential underestimation of the observed effects of neurological diagnosis on eye disease prevalence.

## Conclusion

This cross-sectional study of older adults from a multiethnic Asian population demonstrated that having AD was associated with DR, for which there was limited prior data. These data suggest that AD with diabetes should be followed up more actively by their physicians, with interventions as appropriate, to prevent visual impairments. Potential barriers to care for these vulnerable individuals need to be investigated and addressed in future studies.

## Data Availability Statement

The raw data supporting the conclusions of this article will be made available by the authors, without undue reservation.

## Ethics Statement

The studies involving human participants were reviewed and approved by National Healthcare Group Domain-Specific Review Board protocol number R1500/83/2017. The patients/participants provided their written informed consent to participate in this study.

## Author Contributions

JC, ZZ, CChen, and LS conceived and designed the study. JC, ZZ, DW, BiT, BK, CS, SH, NV, BoT, CCheu, GG, AP-C, TW, CChen, and LS analyzed and interpreted the data. JC, ZZ, and LS wrote the main manuscript text. All authors reviewed the manuscript.

## Funding

This study was supported by the National Medical Research Council (CG/C010A/2017_SERI, OFIRG/0048/2017, OFLCG/004c/2018, TA/MOH-000249-00/2018, MOH-OFIRG20nov-0014, and NMRC/CG2/004b/2022-SERI), National Research Foundation Singapore (NRF2019-THE002-0006 and NRF-CRP24-2020-0001), A^*^STAR (A20H4b0141), the Singapore Eye Research Institute and Nanyang Technological University [SERI-NTU Advanced Ocular Engineering (STANCE) Program], the Duke-NUS Medical School [Duke-NUS-KP(Coll)/2018/0009A], and the SERI-Lee Foundation (LF1019-1) Singapore. The sponsor or funding organization had no role in the design or conduct of this research.

## Conflict of Interest

The authors declare that the research was conducted in the absence of any commercial or financial relationships that could be construed as a potential conflict of interest.

## Publisher's Note

All claims expressed in this article are solely those of the authors and do not necessarily represent those of their affiliated organizations, or those of the publisher, the editors and the reviewers. Any product that may be evaluated in this article, or claim that may be made by its manufacturer, is not guaranteed or endorsed by the publisher.

## Abbreviations

2DD: 2-disc diameters
3MSE: Modified Mini-Mental State Examination
AD: Alzheimer's disease
AMD: age-related macular degeneration
ANOVA: one-way analysis of variance
BMI: body mass index
CI: confidence interval
CIND: cognitive impairment no dementia
DR: diabetic retinopathy
DSM-IV: Diagnostic and Statistical Manual of Mental Disorders
DSST: Digit Symbol Substitution Test
ETDRS: Early Treatment Diabetic Retinopathy Study
MCI: mild cognitive impairment
MMSE: Mini-Mental State Examination
MoCA: Montreal Cognitive Assessment
NCI: no cognitive impairment
NINCDS-ADRDA: National Institute of Neurological and Communicative Disorders and Stroke and the Alzheimer's Disease and Related Disorders Association
NPDR: non-proliferative diabetic retinopathy
OR: odds ratio
SD: standard deviation
VaD: vascular dementia
VEGF: vascular endothelial growth factor.
