# Supplementary material for: Age-Related Eye Diseases in Individuals With Mild Cognitive Impairment and Alzheimer's Disease
Source: Front Aging Neurosci. 2022 Jul 14;14:933853. doi: 10.3389/fnagi.2022.933853 (PMC9329945; doi:10.3389/fnagi.2022.933853)
Supplement: Supplementary file 1 [file Data_Sheet_1.PDF]

Supplementary Material

Supplementary Table 1. Characteristics of participants stratified by gradability of fundus photographs

Characteristics	Gradable (n = 592)	Ungradable (n = 64)	P value*
**Age**, years	72.6 (7.9)	74.8 (7.3)	**0.034**
**Sex**			
Male	260 (43.9)	26 (40.6)	0.614
Female	332 (56.1)	38 (59.4)	
**Ethnicity**			
Chinese	494 (83.4)	54 (84.4)	
Malay	54 (9.1)	6 (9.4)	1.000
Indian	35 (5.9)	4 (6.3)	
Others	9 (1.5)	0 (0.0%)	
**Neurological diagnosis**			
NCI	124 (20.9)	8 (12.5)	**0.008**
CIND	252 (42.6)	20 (31.3)	
AD	216 (36.5)	36 (56.3)	
**Educational level**			
Primary or lower	311 (52.5)	38 (59.4)	0.297
Secondary or higher	281 (47.5)	26 (40.6)	
**Marital status**			
Married	413 (69.8)	39 (60.9)	
Single	31 (5.2)	2 (3.1)	0.243
Divorced	20 (3.4)	2 (3.1)	
Widowed	128 (21.6)	21 (32.8)	
**Living situation**			
Lives with partner/spouse	383 (64.7)	37 (57.8)	
Lives with children/relative/friend	154 (26.0)	20 (31.3)	0.556
Lives alone	31 (5.2)	5 (7.8)	
Others	24 (4.1)	2 (3.1)	
**Smoking**[†]			
Current smoker	42 (7.1)	4 (6.3)	0.972
Ex-smoker	114 (19.3)	13 (20.3)	
Non-smoker	435 (73.5)	46 (71.9)	
**Hyperlipidemia**[‡]			
Yes	420 (70.9)	45 (70.3)	0.884
No	170 (28.7)	19 (29.7)	

**Hypertension**§			
Yes	407 (68.8)	49 (76.6)	0.224
No	181 (30.6)	15 (23.4)	
**Diabetes**			
Yes	194 (32.8)	29 (45.3)	**0.044**
No	398 (67.2)	35 (54.7)	
**Body mass index**‖, kg/m^2	23.9 (4.0)	23.7 (4.0)	0.618
**Systolic blood pressure**¶, mmHg	143.1 (19.3)	141.3 (17.9)	0.472
**Diastolic blood pressure**#, mmHg	73.8 (10.6)	74.7 (12.2)	0.515

Data presented are mean (SD) or number (%), as appropriate.
Abbreviations: NCI, no cognitive impairment; CIND, cognitive impairment, no dementia; AD, Alzheimer's disease.
* P value was obtained with two-tailed independent t-test for the continuous variables and with chi-square test or Fisher's exact test for the categorical variables.
Bold values denote statistical significance at the P < 0.05 level.
† Data available for 591 patients with gradable photographs and 63 patients with ungradable photographs.
‡ Data available for 590 patients with gradable photographs and 64 patients with ungradable photographs.
§ Data available for 588 patients with gradable photographs and 64 patients with ungradable photographs.
‖ Data available for 588 patients with gradable photographs and 59 patients with ungradable photographs.
¶ Data available for 590 patients with gradable photographs and 63 patients with ungradable photographs.
# Data available for 589 patients with gradable photographs and 63 patients with ungradable photographs.

Supplementary Table 2. Association between neurological diagnosis including MCI and DR

	Diabetic retinopathy			
**Neurological diagnosis**	**No. (%)**	**Model 1**	**Model 2**	**Model 3**
NCI	7 (5.6)	Reference	Reference	Reference
CIND without MCI	4 (5.0)	1.07 (0.29-3.88)	1.07 (0.29-3.97)	0.68 (0.17-2.68)
CIND with MCI	9 (5.2)	1.10 (0.39-3.10)	0.99 (0.35-2.82)	0.65 (0.21-1.97)
AD	30 (13.9)	**3.60 (1.45-8.96)**	**2.96 (1.15-7.61)**	1.83 (0.66-5.04)

Abbreviations: AMD, age-related macular degeneration, NCI, no cognitive impairment; CIND, cognitive impairment, no dementia; MCI, mild cognitive impairment; AD, Alzheimer's disease.
Model 1: Adjusted for age, sex, and ethnicity
Model 2: Adjusted for age, sex, ethnicity, educational level, and marital status.
Model 3: Adjusted for age, sex, ethnicity, educational level, marital status, and diabetes.
Bold values denote statistical significance at the $P < 0.05$ level.

Supplementary Table 3. Association between neurological diagnosis and types of AMD

Neurological diagnosis	Early AMD			Intermediate and advanced AMD		
	**No. (%)**	**Model 1**	**Model 2**	**No. (%)**	**Model 1**	**Model 2**
NCI	28 (22.6)	Reference	Reference	45 (36.3)	Reference	Reference
CIND	67 (26.6)	1.07 (0.60-1.92)	1.10 (0.60-1.99)	90 (35.7)	0.90 (0.53-1.51)	0.88 (0.52-1.50)
AD	51 (23.6)	0.80 (0.43-1.48)	0.83 (0.44-1.58)	75 (34.7)	0.73 (0.42-1.28)	0.68 (0.38-1.22)

Abbreviations: AMD, age-related macular degeneration, NCI, no cognitive impairment; CIND, cognitive impairment, no dementia; AD, Alzheimer's disease.
Model 1: Adjusted for age, sex, and ethnicity
Model 2: Adjusted for age, sex, ethnicity, educational level, and marital status.
Bold values denote statistical significance at the $P < 0.05$ level.
